# The oncogenic potential of Rab-like protein 1A (RBEL1A) GTPase: The first review of RBEL1A research with future research directions and challenges

**DOI:** 10.7150/jca.84267

**Published:** 2023-10-02

**Authors:** Ki Lui, Ying Huang, M. Saeed Sheikh, Kwok-Kuen Cheung, Wing Yip Tam, Keng-Ting Sun, Ka Ming Cheng, Winnie Wing Man Ng, Anthony Wai-Keung Loh

**Affiliations:** 1 School of Nursing and Health Studies, Hong Kong Metropolitan University, Hong Kong.; 2 Department of Pharmacology, State University of New York, Upstate Medical University, Syracuse, New York, USA.; 3 Department of Rehabilitation Sciences, The Hong Kong Polytechnic University, Hong Kong.; 4 Department of Surgery, University of British Columbia, Vancouver, British Columbia, Canada.; 5 Division of Medical Sciences & Graduate Entry Medicine, School of Medicine, University of Nottingham, United Kingdom.; 6 School of Nursing, The Hong Kong Polytechnic University, Hong Kong.; 7 Division of Science, Engineering and Health Studies (SEHS), College of Professional and Continuing Education, The Hong Kong Polytechnic University, Hong Kong.

**Keywords:** RBEL1A, GTPase, oncogene, cancer

## Abstract

Research on Rab-like protein 1A (RBEL1A) in the past two decades highlighted the oncogenic properties of this gene. Despite the emerging evidence, its importance in cancer biology was underrated. This is the first RBEL1A critical review covering its discovery, biochemistry, physiological functions, and clinical insights. RBEL1A expression at the appropriate levels appears essential in normal cells and tissues to maintain chromosomal stability; however, its overexpression is linked to tumorigenesis. Furthermore, the upstream and downstream targets of the RBEL1A signaling pathways will be discussed. Mechanistically, RBEL1A promotes cell proliferation signals by enhancing the Erk1/2, Akt, c-Myc, and CDK pathways while blunting the apoptotic signals via inhibitions on p53, Rb, and caspase pathways. More importantly, this review covers the clinical relevance of RBEL1A in the cancer field, such as drug resistance and poor overall survival rate. Also, this review points out the bottle-necks of the RBEL1A research and its future research directions. It is becoming clear that RBEL1A could potentially serve as a valuable target of anticancer therapy. Genetic and pharmacological researches are expected to facilitate the identification and development of RBEL1A inhibitors as cancer therapeutics in the future, which could undoubtedly improve the management of human malignancy.

## A. The discovery of RBEL1A

RBEL1A (Rab-like protein 1A) is also known as Rabl6A (Rab, member of Ras oncogene family-like protein 6A), C9Orf86, FLJ10101, and PARF (Partner of ARF). It is one of the members in the superfamily of Ras GTPases and belongs to Rab (Ras-like protein in the brain) sub-branch based on the protein homology. *RBEL1* gene generates at least four isoforms, RBEL1A, RBEL1B, RBEL1C, and RBEL1D. RBEL1A has received more attention recently as a novel GTPase with oncogenic potential in various human malignancies. This is the first RBEL1A critical review covering its discovery, biochemistry, physiological functions, and clinical insights. Furthermore, the upstream and downstream targets of the RBEL1A signaling pathways and the clinical relevance of RBEL1A studies in oncology will be discussed.

RBEL1A was independently characterized by three groups around the same period 1-3. At first, Lu's group published a short report showing that Esophageal Cancer Related Gene 2 (ECRG2), a tumor suppressor, interacted with a clone FLJ10101 in a yeast two-hybrid library screening 3. The FLJ10101 clone contained an open reading frame of an uncharacterized protein, but the group had not attempted to characterize whether this gene was expressed in cells. Later, our group found that the putative-translated protein of FLJ10101 was identical to RBEL1A. After the ECRG2 report, Quelle's group reported the discovery of PARF (for Partner of ARF; a synonym for RBEL1A) 1. Quelle's group was searching for the p19^ARF^-interacting proteins by using the yeast two-hybrid approach with p19^ARF^ protein as bait, and they identified several p19^ARF^-interacting proteins; one of them was PARF 1. p19^ARF^ is a vital tumor suppressor often deleted or suppressed in cancer 4, 5; hence, identification of PARF-p19^ARF^ interaction shed light on the regulatory roles of RBEL1A on p19^ARF^ tumor suppressor. Although Quelle's group, in their first paper, stated that PARF seemed to be a tumor suppressor protein, they did not further characterize its tumor suppressor function nor its GTPase nature 1.

In about the same period, Huang's group used the computer-based alignment of the Expressed Sequence Tags (ESTs) approach to identify novel proteins dysregulated in human malignancies and/or regulated by stress. One of the open reading frames (ORFs) that they identified contained several conserved regions, namely the “G-motifs,” which were commonly noted in GTPases 2. Further comparison of the novel ORF revealed its N-terminus exhibiting high similarity with the Ras superfamily proteins, specifically, the Rab subfamily proteins. Accordingly, they named the novel protein RBEL1A (for Rab-like protein 1A) 2. Huang's group performed functional characterization of RBEL1A and discovered the GTP-binding potential and its GTPase activity in all isoforms. In their initial study, Huang's group reported two RBEL1 variants, RBEL1A and RBEL1B, which were capable of binding and hydrolyzing GTP 2. Later investigations revealed that the amino acid sequences corresponding to RBEL1A and PARF were identical; thus, RBEL1A discovered by Huang's group, PARF discovered by Quelle's group, and FLJ10101 discovered by Lu's group were essentially the same protein. Furthermore, Huang's group was the first to report the overexpression pattern in cancers and the oncogenic potential of RBEL1A. Their finding was further supported by a robust demonstration of the overexpression pattern of RBEL1A in primary cancer specimens of various human malignancies 2. Subsequent findings also discovered that RBEL1A suppresses p53 tumor suppressor proteins 6. After their original reports, multiple groups have subsequently added experimental evidence to support the notion that RBEL1A is functioning like an oncogenic protein but not a growth suppressor protein, as opposed to the earlier speculation by Quelle's group 1. The key information of RBEL1A-related literature (2003-2022) and the cancer types manifested by RBEL1A overexpression are summarized in Table 1 and 2, respectively. The functional studies of RBEL1A in normal fibroblasts and Schwann cells are also included in Table 2. We will further discuss the functions of RBEL1A, particularly in the cancer field, in the later part of this review.

## B. Sequence analyses

### B1. *RBEL1* gene regulation by promoter, enhancer, and non-coding RNAs

*RBEL1* gene is located on human Chromosome 9q34.3, and its genomic sequence corresponds to 33,266 bases. Although the promoters and enhancers of the *RBEL1* gene have not been characterized experimentally, its regulatory sequences have been analyzed and predicted by the GeneHancer bioinformatics algorithm in the public domain 7. By the *in silico* prediction, the promoter region is predicted to be present at ~1.5 kb upstream from the transcription start site (TSS) and 0.9 kb downstream from the TSS, while the enhancer is 446 kb upstream from the TSS. These cis-acting elements are predicted to be regulated by at least 276 transcription factors and regulatory proteins, such as c-Myc, YY1, FoxM1, Rb, E2F1, E2F5, E4F1, MTA2, HDAC1 & HDAC2, ESR1 (estrogen receptor alpha), ERR1 (estrogen receptor-related receptor alpha), stat1, stat3, and stat5. Many of these transcription factors and regulatory proteins are deregulated in cancer and therefore potentially implicate in the overexpression of RBEL1A gene in cancer malignancies. Yet, the exact mechanisms underlying the regulation of RBEL1A remain to be investigated.

Besides the regulation of gene expression by transcription factors, RBEL1A expression has been demonstrated experimentally being regulated by three non-coding RNAs, i.e. microRNA miR-122-5p (positive regulator) 8, miR-361-3p (negative regulator) 9, and circular RNA circTMC5 (positive regulator) 9. In recent years, growing evidence has shown that non-coding RNAs play an essential role in gene expression regulation, and dysregulation of many of these RNAs lead to diseases, including cancers. Non-coding RNAs can act as tumor suppressors 10, 11, while many others behave as oncogenes 12-17, or some RNA species can be both depending on the circumstances 18. Recently, miR122-5p has been shown to act as an oncogene by targeting the 3' untranslated region (3'UTR) of RBEL1A's mRNA to up-regulate its expression, but this up-regulation can be disrupted by androgen receptor 8. In general, miRNAs targeting mRNA' 3-UTR is believed to block the translation of the mRNA; however, the authors had not further demonstrated how targeting 3'UTR is able to up-regulate RBEL1A's expression. On the other hand, a study has shown that miR-122-5p acts as a tumor suppressor by up-regulating tumor suppressor p53 level, exerting anti-proliferative force to cells 19. It is of interest to determine the interrelationship among them in the RBEL1A-miR122-5p-p53 axis in the future to examine the bifunctional roles of miR122-5p in cells.

Furthermore, recent studies have shown that circular RNAs (circRNAs), a form of closed-loop RNA generated from RNA splicing process, play important roles in regulation of biological processes. Many studies have revealed that circRNAs regulate gene expression by various mechanisms, such as binding to the translation complex and affecting the mRNA turnover rate via sponging (binding) with microRNA, preventing the formation of inhibitory miRNA-mRNA complex 20. Aberrant changes in levels of circRNAs have been linked to carcinogenesis 21. It has been found that circRNA-TMC5 (circTMC5) is overexpressed in gastric cancer and one study 9 has demonstrated that this up-regulation has a significant correlation with the overexpression of RBEL1A in gastric cancer patients' tumor samples. The authors have further elucidated that circTMC5 increases RBEL1A expression by sponging with RBEL1A's inhibitory miRNA-361-3p 9. The up-regulation of RBEL1A in gastric cancer agrees with Huang's previous observation published in 2007 2. As non-coding RNAs apparently become the essential gene regulators, we have attempted to use an open resource computational algorithm CircNet 2.0 to predict a few potential RBEL1A's regulatory miRNAs 22. It shows that miR-1226-3p, miR-425-5p, miR-222-3p, miR-191-5p, miR-10a-5p, miR-484, and let-7e-5p are potential RBEL1A's regulators, and all these miRNAs are up-regulated in the majority of cancers in the database. An up-regulation of these miRNAs, a few if not all, may cause the RBEL1A overexpression in cancers. The ultimate causes of RBEL1A overexpression in cancers warrant further investigation.

### B2. RBEL1 isoforms

**Figure 1:**
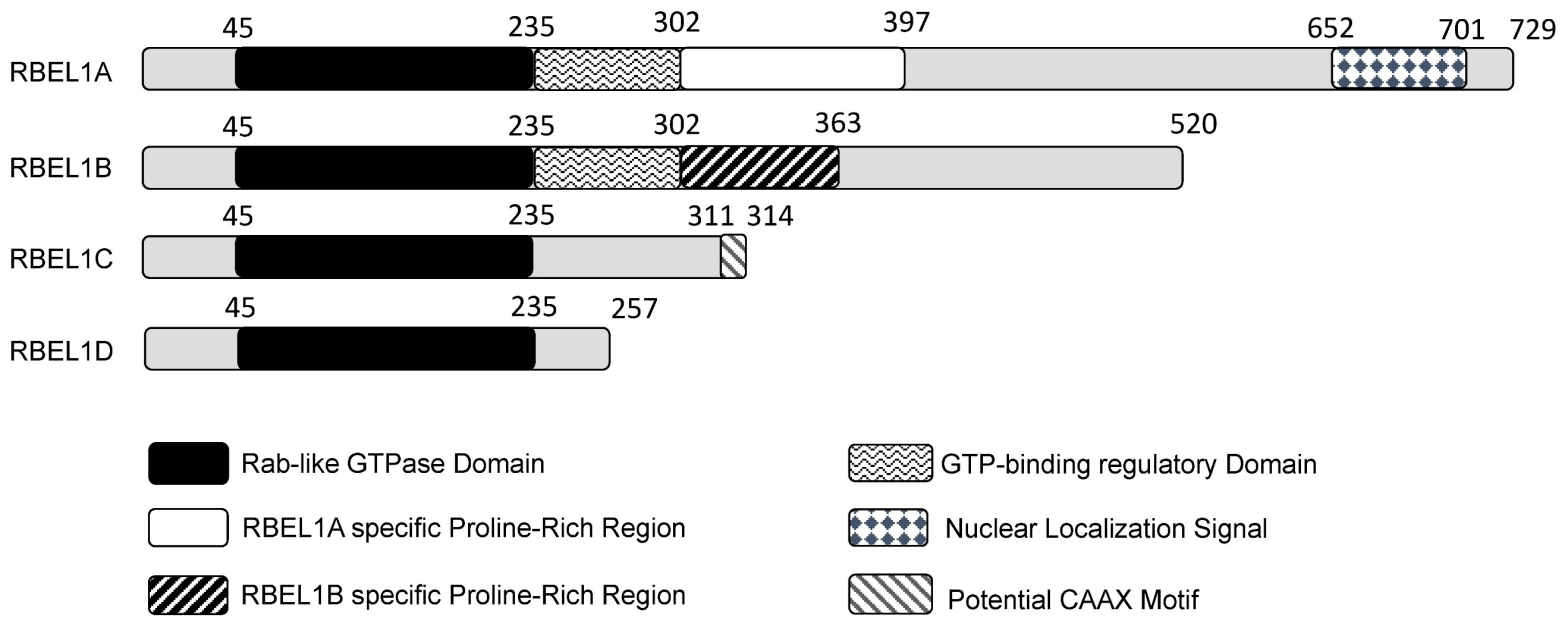
A diagram showing different domains in the four isoforms expressed from the *RBEL1* gene (RBEL1A-D). The number on top of each isoform depicts each domain's amino acid position. Except for the gray regions that do not show specificity, other differently shaded/colored regions represent specific domains within the isoforms.

Huang's group initially reported the identification of two isoforms, they named them RBEL1A and RBEL1B. RBEL1A was longer, whereas RBEL1B was shorter and deficient in some sequences towards the C-terminal side, probably due to alternative RNA splicing. Subsequently, Huang's group reported additional two RBEL1 variants, namely RBEL1C and RBEL1D 23, and altogether all four RBEL1 variants (isoforms A, B, C & D) shared an identical N-terminal GTPase domain but were different at the C-terminal ends (shown in Figure 1). Thus, the *RBEL1* gene expressed four alternatively spliced isoforms, i.e., RBEL1A, RBEL1B, RBEL1C, and RBEL1D 23. The mRNAs of all the *RBEL1* variants contained identical exon 1 to exon 6 but harbored different 3' ends created by different splicing points. Overall, the *RBEL1A* variant comprised 15 exons and was translated to a protein of 729 amino acids (a.a.); *RBEL1B* variant contained 10 exons and was translated to a protein of 520 amino acids, *RBEL1C* variant had 8 exons that coded for a protein of 314 amino acids, and *RBEL1D* variant contained 7 exons that coded for a protein of 257 amino acids. All isoforms, except RBEL1B, could be detected in cancer by immunoblots using the polyclonal antibody that recognizes the antigenic region in the GTPase domain (NH_2_-ALKKLVGSDQAPGRDKN-COOH, 4-20 a.a.). RBEL1A contained a Rab-like GTPase domain (39-235 a.a.) at its amino terminus, followed by a unique proline-rich region (293-617 a.a.), and a nuclear localization signal sequence (NLS, 652-701 a.a.) at its carboxyl terminus. The N-termini of all isoforms shared the identical Rab-like GTPase domain, but their C-termini were different. For example, RBEL1B contained no NLS and harbored a truncated proline-rich region. RBEL1C contained only the GTPase domain with a potential CAAX motif that was predicted to be crucial for the membrane association, while RBEL1D contained the GTPase domain only.

Huang's data showed that RBEL1A was predominantly localized in the cytoplasm with punctate feature while approximately 25% of cells showed nucleocytoplasmic localization 1, 23, but the significance of such distribution remained to be elucidated. This punctate feature of RBEL1A cellular localization pattern somewhat resembled to a few Rab proteins, such as Rab8 and Rab13 24. Although RBEL1A and RBEL1B were both constitutively GTP-bound, RBEL1A was predominantly expressed in cytoplasm while RBEL1B was predominantly expressed in nucleus. More interestingly, a single point mutation at amino acid position 57 (T57N, threonine to asparagine alteration) that disrupted the guanine nucleotide (GTP or GDP) binding in the RBEL1B blocks RBEL1B's nuclear localization, suggesting that the binding of GTP or GDP might affect the RBEL1B's nucleocytoplasmic shuttling. This nucleocytoplasmic shuttling was probably controlled by the GTPase activity via cycling of GTP and GDP binding 2. However, the T57N mutation in RBEL1A did not show significant effect because wild-type RBEL1A was predominantly localized in the cytoplasm. It is still unclear what role does the nucleocytoplasmic shuttling of RBEL1A and RBEL1B play in cells and its regulation mechanism is still unknown.

### B3. RBEL1 GTPase domain and its regulatory region

All RBEL1 isoforms contain a N-terminal GTPase domain which shows high homology to guanine nucleotide (GTP/GDP) binding regions similar to many Rab proteins. Although all RBEL1 isoforms contain identical GTPase domain, it has been experimentally shown that RBEL1A and RBEL1B are predominantly GTP-bound, while RBEL1C and RBEL1D are predominantly GDP-bound 23. The difference in guanine nucleotide binding is mostly influenced by the protein sequence following the GTPase domain. It has been demonstrated that the region at 236-302 a.a. of RBEL1A is crucial for its GTP association, while deletion of this region switches the GTP-binding to GDP-binding. The switching of GDP to GTP binding in a GTPase is known to turn on the protein function from the “OFF” state to the “ON” state in the Ras-family GTPases and vice versa 25, 26. The switching is tightly controlled by two classes of effector proteins, namely nucleotide exchange factors (GEFs) and GTPase activating proteins (GAPs) 27. The GEF proteins facilitate the binding of the GTP (“ON” state) in the GTPase, while the GAP proteins trigger the hydrolysis of the GTP to GDP (“OFF” state) in a GTPase 25, 28, 29. These proteins have been demonstrated to bind with the Switch I and II regions within the GTPase to trigger conformational changes in the guanine-nucleotide binding pocket of the GTPase 30, 31. These two classes of effector proteins are keys to control the switching of the “ON” and “OFF” states of a GTPase 32. However, the GEF and GAP of RBEL1A have not been identified yet; thus, the functional underpinning of the switching between GDP-binding to GTP-binding remains to be explored.

### B4. Polybasic/nuclear localization region

RBEL1A contains a long stretch of basic amino acids (underlined) within position 652-701 a.a. (NH_2_-KEKKKKKKKGKEEEEKAAKKKSKHKKSKDKEEGKEERRRRQ QRPPRSRER-COOH) at its carboxyl terminus, similar to K-Ras isoform B within position 167-184 a.a. (NH_2_-KEKM**S**KDGKKKKKK**S**KTK-COOH). The polybasic region has been shown to be important for membrane association 33. Likewise, our biochemical analysis shows that RBEL1A is loosely associated with membrane 23, similar to many Rab proteins as they direct many membrane trafficking events 34. The RBEL1A polybasic amino acid sequence overlaps with the canonical nuclear localization signal (NLS) within position 652-701 a.a., which has been confirmed to associate with p19^ARF^ 1. RBEL1A is predominantly localized in the cytoplasm; the NLS of RBEL1A is anticipated to contribute to its dynamic shuttling between cytoplasm and nucleus 1, 23.

## C. Physical properties of RBEL1A

### C1. RBEL1A is modified by glycosylation

Glycoproteomic research is a new emerging field in studying the effects of glycan modifications to proteins 35, as it has been shown that glycan modifications affect many protein functions such as protein folding, stability 36, 37, protein subcellular compartment localization 35, and receptor signaling 38, etc. Previously, Huang's group has demonstrated that RBEL1A exists in two forms, the glycosylated and unglycosylated forms, by membrane fractionation assays. The glycosylated-RBEL1A exhibits in the 125-130 kDa size range, whereas the unglycosylated-RBEL1A appears to be in the 95-100 kDa size range when analyzed by immunoblotting using the polyclonal antibody as described in section B2 in this review. They have demonstrated RBEL1A is modified by the *O*-linked *N*-acetylglucosamine glycosylation which is usually attached to serine or threonine amino acid residue on protein 2. Interestingly, Quelle's group has only reported a single RBEL1A form by immunoblot analysis 8, 39, 40. After analyzing all the published data, we provide two possible explanations for such a difference. First, it is possible that different antibodies may have slightly different antigen recognition capabilities that the polyclonal antibody recognizes the two forms, whereas the other antibody detects only one form because the latter one is a monoclonal antibody. Secondly, the two species of RBEL1A might have overlapped in very close proximity in the immunoblot that the two distinct bands could not be recognized, perhaps due to insufficient acrylamide concentration in the separating gels. Smear and multiple band-like signals have sometimes been noted in the raw data in some publications 39, 41-43. Hence, validation of the RBEL1A antibody is particularly important to identify unmodified RBEL1A and modified RBEL1A (glycosylation and other post-translational modifications).

### C2. Weak association of RBEL1A and cell membrane

Canonical Rab protein is crucial for vesicular trafficking processes in which the dynamic of the membrane association and dissociation of Rab protein is controlled by the cycling of the GTP and GDP in the Rab GTPase 28, 32, 44, 45. Moreover, the membrane association of Rab is tightly regulated by GDP Dissociation Inhibitor (GDI) protein, and post-translational modification for example, geranylgeranylation 46. RBEL1A is similar to Rab GTPase in that it is present in both the cytosolic and membrane isolates. Membrane-bound RBEL1A has been shown to loosely associate with cell membrane, while RBEL1B, RBEL1C, and RBEL1D isoforms associate more tightly with the membrane 23. Moreover, only the glycosylated-RBEL1A associates with cell membrane, while the unglycosylated RBEL1A does not, suggesting that the RBEL1A glycosylation may regulate the membrane attachment and detachment cycle 23. However, the functional role of its membrane association still remains unclear. Moreover, the difference in the membrane binding potential between the RBEL1A-GTP and RBEL1A-GDP, and whether RBEL1A is regulated by GDI are questions that warrant further investigations.

## D. Expression and Functional roles of RBEL1A

**Figure 2:**
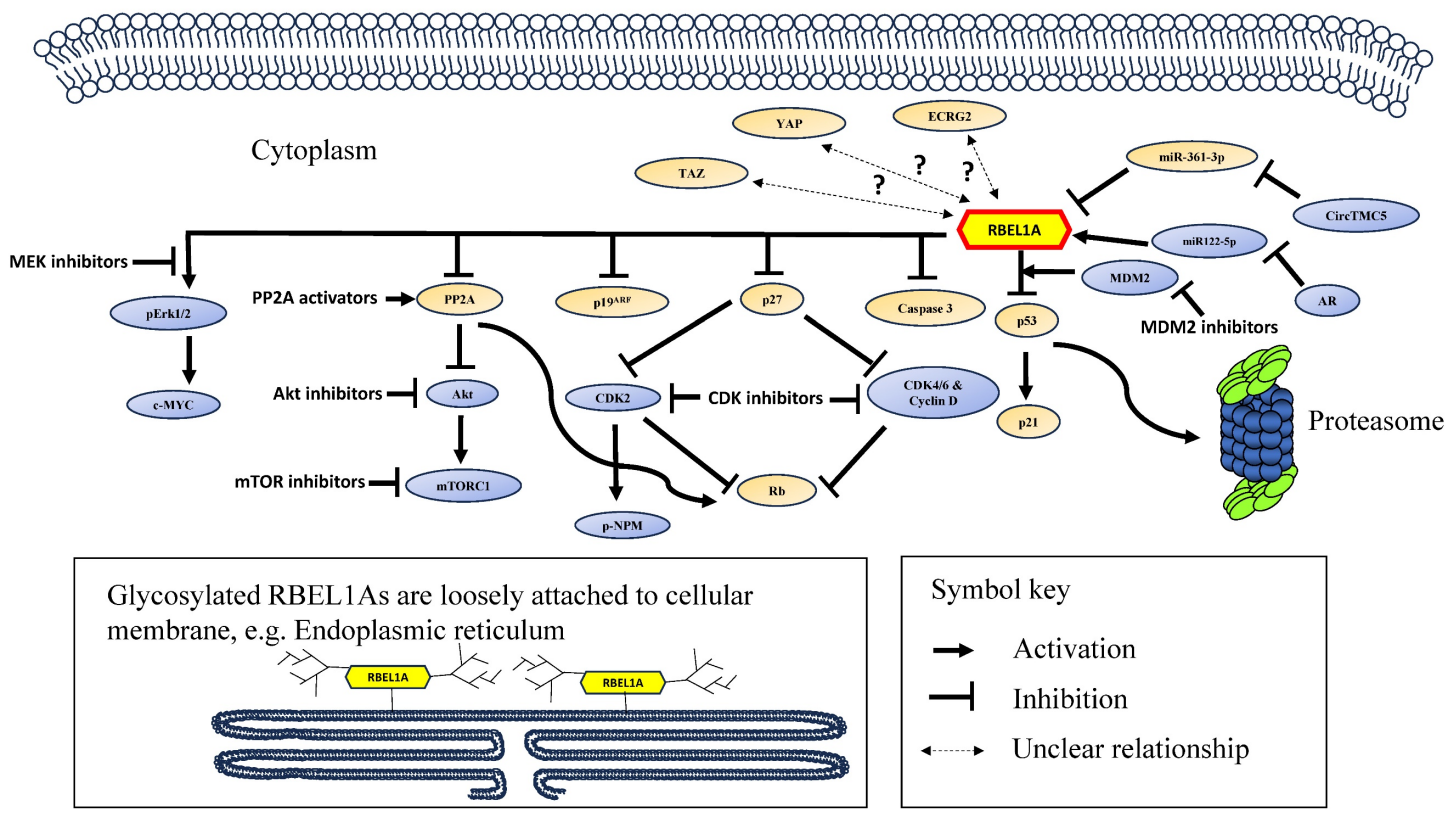
A schematic diagram showing the signaling network of RBEL1A in cancer. RBEL1A plays several key roles in oncogenesis. RBEL1A blocks tumor suppressor p53 by interacting with MDM2 which leads to proteasomal degradation of p53 26, hence it blocks the expression of p21, a p53-regulated cell proliferation inhibitor. Besides MDM2-mediated p53 inhibition, RBEL1A binds to the tetramerization domain of p53 to block the formation of active p53 tetramers 6. Also, RBEL1A blocks the apoptotic caspase 3 activation and the expression of p27, a cyclin-dependent kinase inhibitor. With lower expression of p27, cyclin D and Cyclin-dependent Kinase 2, 4, 6 (CDK2, CDK4, CDK6) are more active, which leads to the inhibition of tumor suppressor Retinoblastoma protein (Rb) 47, facilitating cell cycle progression. Also, it has been found that depletion of RBEL1A leads to CDK2-mediated hyperphosphorylation of nucleophosmin (NPM) in p53 null normal murine fibroblasts, leading to chromosome instability 41. RBEL1A inhibits Phosphatase 2A (PP2A) to increase protein kinase B (Akt) activation, and subsequently to activate Mammalian Target of Rapamycin 1 (mTOR1) protein to promote cell growth 48. Also, PP2A's inhibition attenuates Rb function to facilitate cell cycle progression. RBEL1A activates Extracellular Signal-Regulated Kinase 1/2 (Erk1/2) which leads to activation of c-Myc to enhance proliferation signal. Three non-coding RNAs (two microRNAs and one circular RNA) have been found to regulate RBEL1A expression: (1) miR122-5p increases the expression of RBEL1A 8, but miR122-5p is down-regulated by androgen receptor (AR), leading to decrease in RBEL1A; (2) CircTMC5 up-regulates RBEL1A expression by inhibiting miR-361-3p 9, an inhibitory microRNA to RBEL1A expression. Studies have shown that various small molecule inhibitors, such as inhibitors of MDM2, CDK, AKT, mTOR1, MEK (mitogen-activated extracellular signal-regulated kinase) 42, 49 and PP2A activator 48 are potentially effective in eliminating RBEL1A overexpressing cancers. The glycosylated and unglycosylated RBEL1A are both present in cytoplasm, but only the glycosylated RBEL1A (shown on the lower left) is loosely attached on cellular membrane, such as endoplasmic reticulum. Two RBEL1A interacting proteins (p19^ARF^ 1 and Esophageal Cancer-Related Gene 2 protein (ECRG2) 3) have been identified, RBEL1A inhibits p19^ARF^, but the functional significance of ECRG2-RBEL1A interaction remains unclear. Also, the expressions of the transcription regulator YAP and TAZ have been shown positively linked to RBEL1A in the clinical samples 50, but the mechanism of their molecular interaction is still unknown.

The RBEL1A's functions and its signaling network are summarized in Figure 2 and the major achievements in RBEL1A research have been summarized in Table 3.

RBEL1A protein is widely expressed in many tissues and its homologs are conserved across various species including humans, rhesus monkeys, rodents, chickens, frogs, zebra fishes and mosquitoes. Thus, RBEL1A is an important protein that has been conserved during evolution. It has been shown that RBEL1A is crucial for chromosome stability, because silencing of RBEL1A in normal mouse fibroblasts (MEFs) appears to show aneuploidy, multi-nucleation 41, and senescence 51. Abnormal chromosome duplication has also been detected when RBEL1A and p53 are co-silenced in the MEFs 41. On the contrary, the knockout (KO) mice generated by Quelle's group seem to be viable and fertile in their later studies 52. The group has crossed the RBEL1A KO mice with other types of KO mice to generate double KO mice, such as RIP-Tag2 (RT2) insulinoma model 52, and to generate RBEL1A+Nf1+p53 53 and RBEL1A+Nf1+Cdkn2a triple KO mice 53. They found that pancreatic tumors 52 and malignant peripheral nerve sheath tumors (MPNSTs) 53 developed much more slowly without RBEL1A, suggesting that RBEL1A is important in cancer progression.

RBEL1A is overexpressed in various primary human cancers. For example, Huang's group reported RBEL1A was overexpressed in 67% of breast cancers, 47% of colon cancers and ~21-25% of uterine, ovarian and stomach cancers 2. The overexpression of RBEL1A has also been reported by other laboratories in different malignancies, such as in pancreatic 39, lung 54, bone 47, esophagus 40, liver 8, stomach 9, and neuroendocrine cancers 42. Based on these studies, it is reasonable to suggest that appropriate expression level of RBEL1A is vital for normal tissue homeostasis, but its overexpression could play a promoting role in cancer development and/or progression. These data suggest that cells have to maintain a balanced RBEL1A level, but either too high or too low expression is likely to cause abnormality in cells. To date, a series of results have shown that the RBEL1A overexpression enhances pro-survival signaling pathways and inhibits the tumor suppressor functions as will be discussed in the next sections.

### D1. RBEL1A enhances proliferation and survival signaling

RBEL1A is important in enhancing cell survival by activating pro-survival signaling pathways via manipulating phosphorylation on Erk1/2 and Akt kinases. Overexpression of RBEL1A increases pro-survival Erk1/2 activation via p44/p42 phosphorylations 23. These phosphorylation signals drive cell cycle progressing from G_1_ to S phase, while at the same time inactivating the expression of anti-proliferative genes 55. On the other hand, knockdown of RBEL1A decreases the pro-survival Erk1/2 phosphorylations 43 and increases in cell-cycle inhibitory p27^KIP1^ expression 42, and thus resulting in increase of cell cycle arrest at the G_1_ phase. Moreover, RBEL1A KO cells showed a decreased pro-survival phosphorylation of Akt (Akt-S473) which was caused by an increase in de-phosphorylation activity mediated by protein phosphatase 2A (PP2A) 48. Phosphorylation of Akt (Akt-S473) is known to activate Akt function; on the contrary, de-phosphorylation at the same site by PP2A turns off Akt function, decreasing cell proliferation 56. Knockdown of RBEL1A is also coupled with caspase 3 activation and apoptosis concomitantly 23, 39, 43. It has also been reported that deletion of RBEL1A significantly retarded pancreatic cancer formation in RBEL1A KO mice, possibly due to concurrent down-regulation of pro-survival c-Myc pathway and up-regulation of tumor suppressor p19^ARF^ 52. Quelle's group has further demonstrated the knockdown results, aligning with their KO experiments, that silencing RBEL1A significantly down-regulated c-Myc expression 52. Altogether, RBEL1A is a significant driving force in cell proliferation.

Recently, Quelle's group has generated two MPNST triple gene-knockout models (RBEL1A+Nf1+Cdkn2a and RBEL1A+Nf1+p53) which were created by crossing the RBEL1A KO mice with the double KO mice generated by CRISPR-Cas9 gene editing 53. As expected, they have observed a slower tumor growth in the triple-gene KO models when compared with the controls in which RBEL1A was intact. In addition, they have observed a down-regulation of the YAP oncoprotein, which is a transcription factor dysregulated in Hippo-related pathway in cancer 57. Furthermore, a positive correlation between RBEL1A and YAP expressions has been observed in the sarcoma tissue array containing 163 clinical samples, and these two proteins have been proposed to be controlled under Hippo kinase regulation 50. However, any causality between RBEL1A and Hippo pathway needs to be confirmed. Furthermore, the high c-Myc expression has been detected in tumors coming from RBEL1A+Nf1+Cdkn2a or RBEL1A+Nf1+p53 triple KO mice 53 and this result contradicted with the previous observation of a decrease in c-Myc in RBEL1A knockdown and KO cells 52. Quelle's group has hypothesized that cells could not survive under RBEL1A deletion context, but tumors evolve eventually due to a survival selection pressure to cells which harbor proliferation advantages, such as higher expression of c-Myc, polyploidy and atypia 53. The hypothesis deems further investigation.

### D2. RBEL1A inhibits tumor suppressors-p53 and retinoblastoma (Rb)

RBEL1A has been demonstrated to inhibit two important tumor suppressors, p53 and Rb. It has been shown that RBEL1A physically interacts with p53 and increases its degradation by MDM2-mediated polyubiquitination 26. The GTPase domain of RBEL1A interacts with the tetramerization domain of p53. Both GTP-bound and GDP-bound RBEL1A interact with p53, but the GDP-bound RBEL1A interacts more tightly with p53 26. RBEL1A also negatively regulates p53 function by blocking p53 tetramerization, preventing p53 to form functional tetrameric conformation for transcriptional activities 6. The expression of RBEL1A leading to aberrant function of p53 is also reflected on the downstream effectors of p53, for example on cyclin-dependent kinase inhibitor p21. Such a negative effect on p53 and the downstream effectors can be reversed upon RBEL1A knockdown 26. The available experimental evidence indicates that RBEL1A negatively regulates p53 via two mechanisms, (i) promoting the degradation of p53 and (ii) preventing p53 tetramers formation. As p19^ARF^ also interacts with p53, the interactions amongst RBEL1A-p53-p19^ARF^ in cancer biology are an interesting yet unexplored research area.

RBEL1A down-regulates tumor suppressor Rb by increasing Rb phosphorylation, such hyper-phosphorylation of Rb has been hypothesized to be linked to cancer formation 58. From a mechanistic standpoint, RBEL1A up-regulates cyclin-dependent kinase 2 (CDK2) to achieve Rb hyper-phosphorylation and thereby inhibits Rb tumor suppressor functions. This appears to be another mechanism via which RBEL1A enhances proliferation via deregulation of Rb 42, 43. Knockdown of RBEL1A reverses these effects together with an up-regulation of cyclin-dependent kinase inhibitor p27^KIP1^ coupled with increased apoptosis 42, 43. Collectively, these findings highlight the roles of RBEL1A in deregulation of tumor suppressors via multiple mechanisms.

In addition to its interactions with p53, RBEL1A has been documented to interact with two other proteins namely, p19^ARF^ 1 and ECRG2 3. In the case of its interactions with p19^ARF^, RBEL1A was named the Partner of ARF (PARF) because its carboxyl region comprising 655-693 amino acid residues exhibited direct physical interaction with p19^ARF^ tumor suppressor protein 1. The functional significance of the protein-protein interactions between RBEL1A and p19^ARF^ has been poorly established. Interestingly, Quelle group's initial finding was that silencing of RBEL1A enhanced cell proliferation, possibly via p19^ARF^ pathway, which led the authors to conclude that RBEL1A might be a tumor suppressor 1. However, the authors did not provide experimental evidence to support the tumor suppressor function of RBEL1A. By contrast, several lines of published evidence highlight that RBEL1A functions as an oncogene. Thereafter, Quelle's group has published findings overturning their previous claim and agreeing that RBEL1A is indeed an oncogene.

## E. Clinical relevance of RBEL1A in cancer

### E1. Clinical insights from targeting Ras GTPase as compared to RBEL1A in cancers

Ras GTPase (Ras) is a well-characterized oncogenic protein discovered more than 40 years ago, and on average about 20% of cancer patients possess Ras mutations, while certain cancer types have higher prevalence rate, such as ≈ 90% in pancreatic cancer and ≈ 50% in colon cancer 59. In normal cells, external proliferation signals are transduced by activating the receptor tyrosine kinases (RTKs), leading to the activation of SOS protein (Ras-GEF), subsequently to activate Ras by increasing GTP-bound Ras. It has been found that the GTP-bound: GDP-bound ratio in Ras is remarkably higher in cancer patients with Ras mutation, suggesting that the mutations favor the Ras locked on its GTP-bound form, so that the mutated cells are continuously sending out proliferative signals even without external growth signals, thus cancer formation and progression. Recent findings also showed that overexpression of wild-type Ras could drive the formation of cancer, such as chronic lymphocytic leukemia 60, 61. However, researchers have been struggling to get an actionable Ras inhibitor due to the fact that Ras is a relatively small protein (21KDa) which does not possess deep actionable binding sites for inhibitors to land on it 62. Therefore, therapeutic approaches to target Ras mutations have to be shifted to its upstream (such as TRK inhibitors, SOS inhibitors) and down-stream signaling molecules (such as BRAF inhibitors and Mitogen-activated protein kinase kinase (MEK) inhibitors). RTK inhibitors (such as Lazertinib 63 and Erlotinib 64), anti-EGFR RTK monoclonal antibodies (such as Panitumumab 65 and Cetuximab 66), BRAF inhibitors (such as Vemurafenib, a first FDA-approved inhibitor targeting Ras signaling pathway 67), MEK inhibitors (such as Trametinib 68) are the chemotherapeutic drugs that are currently used to treat cancer patients with Ras mutations. Also, SOS1 inhibitors (such as BI-3406 69, BI 1701963 70, BAY-293 71) and membrane association inhibitors (such as GGTI-2418 72 and FGTI-2734 73) are currently tested in clinical trials with positive preliminary results. After four decades of the discovery of Ras, Sotorasib (AMG510), a Ras targeting drug to inhibit G12C mutation, has been FDA-approved in 2021 74-76. Even previously anticipated that no small molecule was able to bind with Ras, Sotorasib has been made to interact with Ras's P2 pocket to lock it in GDP-bound inactive form 76, demonstrating that small molecule targeting Ras is feasible.

Similar to Ras, RBEL1A has been found to be overexpressed in many cancer types, while some cancers preferentially have higher prevalence rate, such as breast (67%) and colon (47%) cancers. A few signaling pathways have been identified crucial in enhancing RBEL1A-driven cell's proliferation, while blocking these pathways have shown cancer growth inhibition effectively. For example, treatments of CDK inhibitors (Dinaciclib alone, Palbociclib alone or combination) have shown significant tumor growth inhibition in malignant peripheral nerve sheath tumor in-vivo model overexpressing RBEL1A 42. Also, the MK-2206 (AKT inhibitor), Everolimus (mTOR1 inhibitor), small-molecule activator of PP2A (SMAP) have retarded tumor growth in the pancreatic neuroendocrine tumor in vivo model by inhibiting RBEL1A in an Akt-dependent manner 48. Although these drugs have been shown effective in inhibiting tumor growth, they only target the upstream or downstream signaling molecules of RBEL1A, hence they may not be RBEL1A-specific. So, greater side effects arising from these drug treatments are anticipated, leaving the better option for treating RBEL1A-driven cancers is to develop a small molecule that can directly bind and inhibit RBEL1A. As RBEL1A is a relatively larger protein of 150KDa and 90KDa in size, we hypothesize that the number of potential binding sites in RBEL1A protein for small molecule should be higher than that of Ras protein. So, it is feasible to develop an inhibitor of RBEL1A in the future.

### E2. RBEL1A acts as a biomarker for poor clinical outcome of cancers

RBEL1A is overexpressed in multiple cancer types and its overexpression has a significant correlation with poor cancer prognosis and overall survival rate of cancer patients. Table 2 summarizes all the cancer types manifested with the oncogenic RBEL1A and Table 3 has shown the major achievements in RBEL1A studies. All studies point to the direction that RBEL1A is associated to poor cancer prognosis and overall survival of cancer patients. RBEL1A overexpression correlates with poor cancer prognosis and shorter life expectancy in patients having cancers of the breast 77, esophagus 40, pancreas 39 and lung 54, 78. The poor survival rate could be partly due to the development of drug resistance in RBEL1A overexpressing cancers, such as resistance to chemotherapy drugs of cisplatin 79 or oxaliplatin 39. Intriguingly, it has been shown that the development of drug resistance appears partly involved the p53-dependent mechanism 57, 79. The p53-dependent cisplatin drug resistance has also been reported in Ras-driven cancers 80, 81. In RBEL1A overexpressing cancers, the apoptotic signaling of p53 is inhibited as demonstrated by our group, hence it is anticipated that cancer cells still thrive under chemotherapy. From these results, we hypothesize that RBEL1A could serve as a predictive marker of drug resistance in certain chemotherapeutics.

It has also been reported that RBEL1A overexpression correlates with up-regulation of proliferative transcription factor TAZ and YAP expressions in human sarcoma samples, and the authors proposed that all these proteins are regulated under Hippo kinase pathway 50. Dysregulation in Hippo kinase leading to up-regulations of TAZ and YAP is known to contribute to cancer development 82, 83, but the direct link between Hippo pathway and RBEL1A needs further investigation. In addition, a study has indicated that RBEL1A could possibly play a role in the development of aggressive malignant peripheral nerve sheath tumors (MPNSTs) by increasing mutations in Nf1, a gene that forms neurofibromin which is crucial in nervous tissues to maintain a balanced survival signal in neurons 53. Nf1 regulates a number of cell signaling pathways, including Ras, MAPK, AKT, mTOR, cAMP, etc 84. Nf1 is regarded as a tumor suppressor as it switches off cell proliferation signal from Ras by its GAP function, thus dysregulation of Nf1 likely plays a role in cancer formation 85. Altogether, we hypothesize that RBEL1A overexpression appears to be a marker of aggressive cancers.

RBEL1A is overexpressed in human malignancy, and it deregulates important signaling pathways for example, those controlled by p53, Erk1/2, CDK-Rb that linked to tumorigenesis. Given its oncogenic role, we hypothesize that RBEL1A could potentially serve as a valuable target of anti-cancer therapy. In this context, Idasanutlin, a potential drug that can rescue p53 function by blocking the inhibitory counterpart MDM2, can be used in the RBEL1A overexpressing cancers as a therapeutic 86. *Melicope ptelefolia* herbal extract can decrease *RBEL1A* gene expression in colon and liver cancer cells, suggesting that certain compound(s) in this herb is/are able to inhibit RBEL1A expression in cancers 87 and could be developed as RBEL1A-targeted therapeutic(s). Because RBEL1A knockdown inhibits cancer cell growth as well as trigger cell death 23, 26, we hypothesize that silencing *RBEL1A* gene expression by small molecule may yield anti-cancer effect. Approaches such as CRISPR/Cas9 technology can be further refined to achieve robust silencing of RBEL1A to target it in human cancers. Recently, palbociclib, a CDK4/6 inhibitor, has been reported to inhibit the tumor growth in RBEL1A overexpressing MPNSTs 42, 88. It will be desirable to explore the effects of Palbociclib on RBEL1A in RBEL1A overexpressing tumors.

## F. Bottle-necks of the RBEL1A research

A number of critical questions about RBEL1A remain unanswered in the RBEL1A research. Without such key information, the RBEL1A research is bottle necked.

1. *Does the ratio of GTP-bound:GDP-bound RBEL1A have any functional implication in cancer cells? What are the RBEL1A's GEF and GAP proteins?* Huang's data have shown that RBEL1A is highly expressed in cancers, which is predominantly (>95%) GTP-bound in cancer cells. However, it is still uncertain whether the ratio of GTP-bound:GDP-bound RBEL1A yields difference in term of the intensity of RBEL1A-driven proliferative signal. Based on the canonical model of Ras GTPases, RBEL1A is locked in its constitutively GTP-bound active form to exert proliferative signals to cells and it can only be assumed that a higher ratio of GTP-bound:GDP-bound RBEL1A results in a higher cellular proliferative activity. If such an assumption is valid, the following question to be asked is which proteins could influence the GTP/GDP binding in RBEL1A. However, the regulation of the GTP/GDP binding of RBEL1A is still not clear. It is still unknown which proteins are RBEL1A's GAP proteins to turn off its function by enhancing its GTP hydrolysis in RBEL1A. Likewise, it is unknown which proteins are RBEL1A's GEF proteins to turn on its function by increasing the exchange of GDP to GTP in the RBEL1A GTPase pocket. Hence, researchers are unable to turn off RBEL1A's functions by locking it in its GDP-bound (inactive) form. Similar to Ras signaling, it is expected that blocking the guanine nucleotide exchange process in the GTPase is able to block RBEL1A's function. For example, inhibitors of SOS proteins (Ras's GEF protein) have been demonstrated to inhibit Ras functions in cells and clinical trials 69-71. It is possible that blocking RBEL1A's GEF-RBEL1A interaction may yield a similar therapeutic outcome. The area of the regulation of the GTPase activity in RBEL1A is still unexplored, and the identification of the RBEL1A's GEF and GAP proteins is urgently needed to allow direct elucidation of the RBEL1A's functions.

*2. What are the post-translational modifications (PTMs) of RBEL1A and their functional significance?* The functions of Ras and all other cellular proteins can be regulated by various mechanisms, such as protein PTMs including phosphorylation, ubiquitination, acetylation, etc 89. For example, phosphorylation of Ras by SRC kinases on tyrosine position 32 and 64 enhances Ras-GAP protein binding to deactivate Ras function 90, 91. Protein Kinase C (PKC) phosphorylates Ras (KRAS4B) on serine 181 site which is known to block Ras's function by inhibiting the membrane association 92. Identification of the post-translational modifications of RBEL1A allows further understanding of the RBEL1A regulation. In addition, targeting the specific PTMs allows precise control of the protein functions, for example treating cancer cells with small molecules that target specific PTM for driving proliferative signal is a novel therapeutic approach in future precision medicine 93. So far, RBEL1A is known to be modified by glycosylation 23, but other RBEL1A's PTM and its downstream signaling molecules are completely unknown. Hence, RBEL1A's PTM data are another pieces of crucial information that are missing and should be collected for the RBEL1A research.

*3. What is the protein three-dimensional (3D) structure of RBEL1A?* The information of the three-dimensional structure of a protein provides key computational and experimental insights about its biologic activities 94. For example, the difference in the crystal structures of Ras in GTP-bound (active) and GDP-bound (inactive) forms have demonstrated the conformational changes in the P-loop structures during the activation, providing biochemical detailed explanations about how the signals are transduced to its downstream targets 95, such as the interaction interface between Ras and BRAF, its downstream target, to elucidate the BRAF activation mechanism 96. Furthermore, it allows inhibitor design that utilizes the 3D-model of the Ras structure to show the docking of the molecules on the protein 97. Thus, obtaining the protein crystal structure of RBEL1A is highly desirable in RBEL1A research. However, the protein of RBEL1A is a lot more difficult to crystalize than Ras due to the fact that the unglycosylated RBEL1A (90KDa) is ≈ 4 times larger than Ras (21KDa) in size. Our group has attempted to crystalize the full-length as well as the GTPase domain of RBEL1A in the past, but most proteins fell into the insoluble inclusion body. Until recent years, Alpha-Fold, a computational algorithm has been launched to allow structural prediction by using the protein sequence alone 98. Although the structure of RBEL1A GTPase domain (refer to Figure 1, region in black) can be predicted with high confidence score, the remaining two-third of the structures in the RBEL1A regulatory regions could not be predicted, again failing to provide further RBEL1A structural insight. Thus, the 3D structure of RBEL1A is another key missing information that deserves further research.

## G. Summary and future directions

In summary, several lines of published evidence indicate that RBEL1A is a protein that possesses oncogenic properties. RBEL1A is overexpressed in human cancers that emerges to predict aggressive malignancy and anticancer drug resistance. Thus, RBEL1A could be a potential biomarker of cancer prognosis and efficacy of chemotherapeutics. The identification of the GEF and GAP regulators of RBEL1A, the post-translational modifications of RBEL1A, the 3D protein structure of RBEL1A will likely speed up the RBEL1A research. Further dissection of the complex wiring of the RBEL1A signaling will provide a clearer picture of oncogenic roles linked to RBEL1A. Finally, the identification and development of RBEL1A inhibitors (e.g., small molecules or herbal extracts) as cancer therapeutics will likely improve the management of human malignancies.

## Figures and Tables

**Table 1:** The emerging roles of RBEL1A in cancer biology (A) and general biology (B) reported in the past two decades.

Year	Key functions/findings of RBEL1A	Reference(s)
**A. Cancer-related field**		
2003	FLJ10101 (RBEL1A) interacts with Esophageal Cancer Related Gene 2 (ECRG2) in a yeast two hybrid screening.	3
2006	PARF (RBEL1A) interacts with p19^ARF^ in a yeast two hybrid screening.	1
2007	RBEL1A and RBEL1B isoforms have been cloned and characterized. RBEL1A is overexpressed in cancer.	2
2009	RBEL1A increases Erk1/2 survival signaling while its knockdown increases caspase 3 activation to induce apoptosis in breast cancer.	23
2013	RBEL1A inhibits p53 by increase in MDM2-mediated proteasomal degradation.	26
	RBEL1A is crucial in centrosome regulation and chromosomal stability in mouse p19^ARF^ (p14^ARF^ is the human ortholog) and p53 independent manner.	41
	High levels of RBEL1A act as a biomarker in breast cancer patients, while down-regulation of RBEL1A prohibits cell proliferation, invasion and cancer growth.	77
	RBEL1A increases resistance to Oxaliplatin treatment in pancreatic cancer.	39
2014	RBEL1A increases pancreatic cancer cell proliferation by promoting G1-S phase transition in a Rb1-dependent manner.	43
2015	RBEL1A inhibits p53 oligomerization via interaction with p53 tetramerization domain.	6
2016	C9Orf86 (RBEL1A) expression is a poor prognosis marker for non-small cell lung cancer.	54, 78
	RBEL1 (RBEL1A) increases cell proliferation of osteosarcoma by inhibiting Rb.	47
2018	RBEL1A is up-regulated by cisplatin and inhibits p53 transcription activity leading to decrease in chemosensitivity of cancer to the drug treatment.	79
	Treatment of *Melicope ptelefolia* leaf extract with the 2 cancer cell lines (HCT116 colorectal cancer and HepG2 hepatocellular cancer) shows down-regulation of RBEL1A and concomitantly up-regulation of p53 gene expressions.	87
2019	RBEL1A suppresses PP2A tumor suppressor and activates AKT signaling in pancreatic cancer.	48
2020	RBEL1A drives the formation of malignant peripheral nerve sheath tumors (MPNST) in Rb-dependent manner. But, this type of cancer is sensitive to treatment of CDK4/6 inhibitors.	88
	Up regulation of RBEL1A mRNA and proteins is found in human esophageal cancer biopsies, which correlates with poor prognosis.	40
2021	High RBEL1A expression in sarcoma patients shows a faster metastatic rate. RBEL1A is linked to p53-MDM2 axis and potentially regulated by Hippo pathway involving YAP and TAZ.	50
	RBEL1A increases Schwann cell's proliferation by regulating Rb.	51
	Loss of RBEL1A expression in RIP-Tag2 (RT) insulinoma mouse model shows a slower progression of pancreatic tumor formation and angiogenesis, suggesting RBEL1A is crucial in cancer proliferation and angiogenesis.	52
	Combination of CDK4/6 inhibitor and Rb inhibition may achieve therapeutic outcome to treat malignant peripheral nerve sheath tumors that overexpress RBEL1A.	49
	Expression of androgen receptor in hepatocarcinoma reduces RBEL1A expression which in turn suppresses invasion and migration. Androgen receptor inhibits RBEL1A's activating miRNA miR-122-5p.	8
2022	RBEL1A drives progression of NF1-associated malignant peripheral nerve sheath tumors.	53
	Circular RNA (circTMC5) is up-regulated in gastric cancer, leading to down-regulation of miR361-3p, subsequently up-regulation of RBEL1A and inhibition of apoptosis. RBEL1A regulates immune cells and their infiltration processes.	9

**B. Other fields**		
2011	Mutation in C9orf86 (RBEL1A) is a candidate gene for recessive cognitive disorders.	99
2015	High RBEL1A protein level is detected in patients' sera infected with Knowlesi malaria.	100
2016	RBEL1A is not a cause of the early onset of rare development disorder in brain.	101
2016	RBEL1A knockdown increases bacterial infection of HT-29 cells by T3SS2 V. parahaemolyticus through affecting the sulfation in a CRISPR/Cas9 screen.	102

**Table 2:** A table showing various cancer types and normal tissues/cells that RBEL1A regulates.

Cancer type	References
Breast	2, 6, 26, 77, 79
Colon	2
Esophagus	40
Liver	8
Lung	54, 78
Stomach	9
Osteosarcoma/sarcoma	50
Pancreas	39, 43, 52, 103
Neuronal (peripheral nerve sheath tumors)	49, 53, 88
**Normal tissue type**	**References**
fibroblasts	41
Schwann cells	51

**Table 3:** A table showing the main achievements of RBEL1A's studies.

Major achievements	References
1. RBEL1A was overexpressed and up-regulated in various types of cancer and it plays a role in carcinogenesis.	2, 40, 42, 43, 47, 48, 52, 77
2. Higher RBEL1A expressing cancers were more resistant to chemotherapy. RBEL1A expression was a poor-prognosis marker for cancer patients.	39, 40, 54, 78, 79
2. RBEL1A was linked to the regulation of tumor suppressor p53. RBEL1A inhibited tumor suppressor protein p53 via two mechanisms: (i) RBEL1A interacted with MDM2 to increase p53 degradation. (ii) RBEL1A interacted with p53 tetramerization domain to prevent p53 tetramer formation.	6, 26, 50, 79, 87
3. RBEL1A blocked tumor suppressor retinoblastoma (Rb) function.	42, 49
4. RBEL1A increased cancer cell proliferation via an increase in phosphorylation of Erk1/2, and subsequently activation of c-Myc. Also, RBEL1A increased Akt phosphorylation and subsequently activation of mTOR1.	23, 48, 52
5. RBEL1A's expression was regulated by three non-coding RNAs (2 microRNAs and 1 circular RNA): miR122-5p and CircTMC5 up-regulate RBEL1A, while miR361-3p down-regulates RBEL1A.	8, 9

## References

[1] Tompkins V, Hagen J, Zediak VP, Quelle DE (2006). Identification of novel ARF binding proteins by two-hybrid screening. Cell Cycle.

[2] Montalbano J, Jin W, Sheikh MS, Huang Y (2007). RBEL1 is a novel gene that encodes a nucleocytoplasmic Ras superfamily GTP-binding protein and is overexpressed in breast cancer. J Biol Chem.

[3] Cui YP, Wang JB, Zhang XY, Bi MX, Guo LP, Lu SH (2003). Using yeast two-hybrid system to identify ECRG2 associated proteins and their possible interactions with ECRG2 gene. World J Gastroenterol.

[4] Pomerantz J, Schreiber-Agus N, Liégeois NJ, Silverman A, Alland L, Chin L (1998). The Ink4a tumor suppressor gene product, p19Arf, interacts with MDM2 and neutralizes MDM2's inhibition of p53. Cell.

[5] Inoue K, Fry EA (2018). Aberrant Expression of p14(ARF) in Human Cancers: A New Biomarker?. Tumor Microenviron.

[6] Lui K, Sheikh MS, Huang Y (2015). Regulation of p53 oligomerization by Ras superfamily protein RBEL1A. Genes Cancer.

[7] Fishilevich S, Nudel R, Rappaport N, Hadar R, Plaschkes I, Iny Stein T (2017). GeneHancer: genome-wide integration of enhancers and target genes in GeneCards. Database (Oxford). 2017.

[8] Tang N, Dou X, You X, Li Y, Li X, Liu G (2021). Androgen Receptors Act as a Tumor Suppressor Gene to Suppress Hepatocellular Carcinoma Cells Progression via miR-122-5p/RABL6 Signaling. Front Oncol.

[9] Xu P, Xu X, Wu X, Zhang L, Meng L, Chen Z (2022). CircTMC5 promotes gastric cancer progression and metastasis by targeting miR-361-3p/RABL6. Gastric Cancer.

[10] Li Z, Ruan Y, Zhang H, Shen Y, Li T, Xiao B (2019). Tumor-suppressive circular RNAs: Mechanisms underlying their suppression of tumor occurrence and use as therapeutic targets. Cancer Sci.

[11] Otmani K, Lewalle P (2021). Tumor Suppressor miRNA in Cancer Cells and the Tumor Microenvironment: Mechanism of Deregulation and Clinical Implications. Front Oncol.

[12] Wang KW, Dong M (2019). Role of circular RNAs in gastric cancer: Recent advances and prospects. World J Gastrointest Oncol.

[13] Chaichian S, Shafabakhsh R, Mirhashemi SM, Moazzami B, Asemi Z (2020). Circular RNAs: A novel biomarker for cervical cancer. J Cell Physiol.

[14] Hao S, Cong L, Qu R, Liu R, Zhang G, Li Y (2019). Emerging roles of circular RNAs in colorectal cancer. Onco Targets Ther.

[15] Shabaninejad Z, Vafadar A, Movahedpour A, Ghasemi Y, Namdar A, Fathizadeh H (2019). Circular RNAs in cancer: new insights into functions and implications in ovarian cancer. J Ovarian Res.

[16] Peng Y, Croce CM (2016). The role of MicroRNAs in human cancer. Signal Transduct Target Ther.

[17] Azimi M, Totonchi M, Ebrahimi M (2022). Determining The Role of MicroRNAs in Self-Renewal, Metastasis and Resistance to Drugs in Human Gastric Cancer Based on Data Mining Approaches: A Systematic Review. Cell J.

[18] Faramin Lashkarian M, Hashemipour N, Niaraki N, Soghala S, Moradi A, Sarhangi S (2023). MicroRNA-122 in human cancers: from mechanistic to clinical perspectives. Cancer Cell Int.

[19] Li KW, Wang SH, Wei X, Hou YZ, Li ZH (2020). Mechanism of miR-122-5p regulating the activation of PI3K-Akt-mTOR signaling pathway on the cell proliferation and apoptosis of osteosarcoma cells through targeting TP53 gene. Eur Rev Med Pharmacol Sci.

[20] Kristensen LS, Andersen MS, Stagsted LVW, Ebbesen KK, Hansen TB, Kjems J (2019). The biogenesis, biology and characterization of circular RNAs. Nat Rev Genet.

[21] Huang JS, Yu SH, Ding L, Ma LY, Chen HJ, Zhou H (2021). The Dual Role of Circular RNAs as miRNA Sponges in Breast Cancer and Colon Cancer. Biomedicines.

[22] Chen Y, Yao L, Tang Y, Jhong JH, Wan J, Chang J (2022). CircNet 2.0: an updated database for exploring circular RNA regulatory networks in cancers. Nucleic Acids Res.

[23] Montalbano J, Lui K, Sheikh MS, Huang Y (2009). Identification and characterization of RBEL1 subfamily of GTPases in the Ras superfamily involved in cell growth regulation. J Biol Chem.

[24] Li H, Ou L, Fan J, Xiao M, Kuang C, Liu X (2017). Rab8A regulates insulin-stimulated GLUT4 translocation in C2C12 myoblasts. FEBS Lett.

[25] Simanshu DK, Nissley DV, McCormick F (2017). RAS Proteins and Their Regulators in Human Disease. Cell.

[26] Lui K, An J, Montalbano J, Shi J, Corcoran C, He Q (2013). Negative regulation of p53 by Ras superfamily protein RBEL1A. J Cell Sci.

[27] Barr F, Lambright DG (2010). Rab GEFs and GAPs. Curr Opin Cell Biol.

[28] Barr FA (2013). Review series: Rab GTPases and membrane identity: causal or inconsequential?. J Cell Biol.

[29] Ishida M, M EO, Fukuda M (2016). Multiple Types of Guanine Nucleotide Exchange Factors (GEFs) for Rab Small GTPases. Cell Struct Funct.

[30] Stenmark H (2009). Rab GTPases as coordinators of vesicle traffic. Nat Rev Mol Cell Biol.

[31] Toma-Fukai S, Shimizu T (2019). Structural Insights into the Regulation Mechanism of Small GTPases by GEFs. Molecules.

[32] Lui K, Huang Y (2009). RanGTPase: A Key Regulator of Nucleocytoplasmic Trafficking. Mol Cell Pharmacol.

[33] Dharmaiah S, Bindu L, Tran TH, Gillette WK, Frank PH, Ghirlando R (2016). Structural basis of recognition of farnesylated and methylated KRAS4b by PDEdelta. Proc Natl Acad Sci U S A.

[34] Jin H, Tang Y, Yang L, Peng X, Li B, Fan Q (2021). Rab GTPases: Central Coordinators of Membrane Trafficking in Cancer. Front Cell Dev Biol.

[35] Moremen KW, Tiemeyer M, Nairn AV (2012). Vertebrate protein glycosylation: diversity, synthesis and function. Nat Rev Mol Cell Biol.

[36] Bektas M, Rubenstein DS (2011). The role of intracellular protein O-glycosylation in cell adhesion and disease. J Biomed Res.

[37] Wandall HH, Nielsen MAI, King-Smith S, de Haan N, Bagdonaite I (2021). Global functions of O-glycosylation: promises and challenges in O-glycobiology. Febs j.

[38] Zhang J, Ten Dijke P, Wuhrer M, Zhang T (2021). Role of glycosylation in TGF-β signaling and epithelial-to-mesenchymal transition in cancer. Protein Cell.

[39] Muniz VP, Askeland RW, Zhang X, Reed SM, Tompkins VS, Hagen J (2013). RABL6A Promotes Oxaliplatin Resistance in Tumor Cells and Is a New Marker of Survival for Resected Pancreatic Ductal Adenocarcinoma Patients. Genes Cancer.

[40] Feng Y, Yan S, Huang Y, Huang Q, Wang F, Lei Y (2020). High expression of RABL6 promotes cell proliferation and predicts poor prognosis in esophageal squamous cell carcinoma. BMC Cancer.

[41] Zhang X, Hagen J, Muniz VP, Smith T, Coombs GS, Eischen CM (2013). RABL6A, a novel RAB-like protein, controls centrosome amplification and chromosome instability in primary fibroblasts. PLoS One.

[42] Kohlmeyer JL, Kaemmer CA, Pulliam C, Maharjan CK, Samayoa AM, Major HJ (2020). RABL6A Is an Essential Driver of MPNSTs that Negatively Regulates the RB1 Pathway and Sensitizes Tumor Cells to CDK4/6 Inhibitors. Clin Cancer Res.

[43] Hagen J, Muniz VP, Falls KC, Reed SM, Taghiyev AF, Quelle FW (2014). RABL6A promotes G1-S phase progression and pancreatic neuroendocrine tumor cell proliferation in an Rb1-dependent manner. Cancer Res.

[44] Borchers AC, Langemeyer L, Ungermann C (2021). Who's in control? Principles of Rab GTPase activation in endolysosomal membrane trafficking and beyond. J Cell Biol.

[45] Hutagalung AH, Novick PJ (2011). Role of Rab GTPases in membrane traffic and cell physiology. Physiol Rev.

[46] Edler E, Stein M (2019). Recognition and stabilization of geranylgeranylated human Rab5 by the GDP Dissociation Inhibitor (GDI). Small GTPases.

[47] Tang H, Ji F, Sun J, Xie Y, Xu Y, Yue H (2016). RBEL1 is required for osteosarcoma cell proliferation via inhibiting retinoblastoma 1. Mol Med Rep.

[48] Umesalma S, Kaemmer CA, Kohlmeyer JL, Letney B, Schab AM, Reilly JA (2019). RABL6A inhibits tumor-suppressive PP2A/AKT signaling to drive pancreatic neuroendocrine tumor growth. J Clin Invest.

[49] Kohlmeyer JL, Gordon DJ, Tanas MR, Dodd RD, Monga V, Darbro BW (2021). Combination therapies for MPNSTs targeting RABL6A-RB1 signaling. Oncotarget.

[50] Desai C, Thomason J, Kohlmeyer JL, Reisetter AC, Ahirwar P, Jahanseir K (2021). Prognostic and therapeutic value of the Hippo pathway, RABL6A, and p53-MDM2 axes in sarcomas. Oncotarget.

[51] Kohlmeyer JL, Kaemmer CA, Umesalma S, Gourronc FA, Klingelhutz AJ, Quelle DE (2021). RABL6A Regulates Schwann Cell Senescence in an RB1-Dependent Manner. Int J Mol Sci.

[52] Maharjan CK, Umesalma S, Kaemmer CA, Muniz VP, Bauchle C, Mott SL (2021). RABL6A Promotes Pancreatic Neuroendocrine Tumor Angiogenesis and Progression In Vivo. Biomedicines.

[53] Kohlmeyer JL, Kaemmer CA, Lingo JJ, Voigt E, Leidinger MR, McGivney GR (2022). Oncogenic RABL6A promotes NF1-associated MPNST progression in vivo. Neurooncol Adv.

[54] Yoshimura K, Osman M, Inoue Y, Suda T, Sugimura H (2016). A novel prognostic marker of non-small cell lung cancer: chromosome 9 open reading frame 86 (C9orf86). J Thorac Dis.

[55] Meloche S, Pouyssegur J (2007). The ERK1/2 mitogen-activated protein kinase pathway as a master regulator of the G1- to S-phase transition. Oncogene.

[56] Franke TF (2008). PI3K/Akt: getting it right matters. Oncogene.

[57] Cunningham R, Hansen CG (2022). The Hippo pathway in cancer: YAP/TAZ and TEAD as therapeutic targets in cancer. Clin Sci (Lond).

[58] Dick FA, Rubin SM (2013). Molecular mechanisms underlying RB protein function. Nat Rev Mol Cell Biol.

[59] Prior IA, Hood FE, Hartley JL (2020). The Frequency of Ras Mutations in Cancer. Cancer Res.

[60] Hortal AM, Oeste CL, Cifuentes C, Alcoceba M, Fernandez-Pisonero I, Clavain L (2022). Overexpression of wild type RRAS2, without oncogenic mutations, drives chronic lymphocytic leukemia. Mol Cancer.

[61] Sheffels E, Kortum RL (2021). The Role of Wild-Type RAS in Oncogenic RAS Transformation. Genes-Basel.

[62] Moore AR, Rosenberg SC, McCormick F, Malek S (2020). RAS-targeted therapies: is the undruggable drugged?. Nat Rev Drug Discov.

[63] Cho BC, Ahn MJ, Kang JH, Soo R, Baisamut T, Yang JCH (2022). A randomized, double-blind, multinational phase III study to assess the efficacy and safety of lazertinib versus gefitinib in the first-line treatment of patients with EGFR mutation (EGFRm), advanced NSCLC (LASER301; NCT04248829). Ann Oncol.

[64] Aran V, Omerovic J (2019). Current Approaches in NSCLC Targeting K-RAS and EGFR. International Journal of Molecular Sciences.

[65] Thibault C, Taieb PJ (2013). Panitumumab-FOLFOX4 Treatment and RAS Mutations in Colorectal Cancer. Oncologie.

[66] Tsai HL, Lin CC, Sung YC, Chen SH, Chen LT, Jiang JK The emergence of RAS mutations in patients with RAS wild-type mCRC receiving cetuximab as first-line treatment: a noninterventional, uncontrolled multicenter study. Br J Cancer. 2023: 10.1038/s41416-023-02366-z. Advance online publication.

[67] Callahan MK, Rampal R, Harding JJ, Klimek VM, Chung YR, Merghoub T (2012). Progression of RAS-Mutant Leukemia during RAF Inhibitor Treatment. New Engl J Med.

[68] Decroocq J, Birsen R, Montersino C, Chaskar P, Mano J, Poulain L (2022). RAS activation induces synthetic lethality of MEK inhibition with mitochondrial oxidative metabolism in acute myeloid leukemia. Leukemia.

[69] Hofmann MH, Gmachl M, Ramharter J, Savarese F, Gerlach D, Marszalek JR (2021). BI-3406, a Potent and Selective SOS1-KRAS Interaction Inhibitor, Is Effective in KRAS-Driven Cancers through Combined MEK Inhibition. Cancer Discov.

[70] Hofmann MH, Lu HY, Duenzinger U, Gerlach D, Trapani F, Machado AA (2021). Trial in Process: Phase 1 studies of BI 1701963, a SOS1::KRAS Inhibitor, in combination with MEK inhibitors, irreversible KRASG12C inhibitors or irinotecan. Cancer Research.

[71] Plangger A, Rath B, Stickler S, Hochmair M, Lang C, Weigl L (2022). Cytotoxicity of combinations of the pan-KRAS SOS1 inhibitor BAY-293 against pancreatic cancer cell lines. Discov Oncol.

[72] Karasic TB, Chiorean EG, Sebti SM, O'Dwyer PJ (2019). A Phase I Study of GGTI-2418 (Geranylgeranyl Transferase I Inhibitor) in Patients with Advanced Solid Tumors. Target Oncol.

[73] Kazi A, Xiang S, Yang H, Chen L, Kennedy P, Ayaz M (2019). Dual Farnesyl and Geranylgeranyl Transferase Inhibitor Thwarts Mutant KRAS-Driven Patient-Derived Pancreatic Tumors. Clin Cancer Res.

[74] Kwan AK, Piazza GA, Keeton AB, Leite CA (2022). The path to the clinic: a comprehensive review on direct KRAS(G12C) inhibitors. J Exp Clin Cancer Res.

[75] Hong DS, Fakih MG, Strickler JH, Desai J, Durm GA, Shapiro GI (2020). KRAS(G12C) Inhibition with Sotorasib in Advanced Solid Tumors. Oncologie.

[76] Liu J, Kang R, Tang D (2022). The KRAS-G12C inhibitor: activity and resistance. Cancer Gene Ther.

[77] Li YY, Fu S, Wang XP, Wang HY, Zeng MS, Shao JY (2013). Down-regulation of c9orf86 in human breast cancer cells inhibits cell proliferation, invasion and tumor growth and correlates with survival of breast cancer patients. PLoS One.

[78] Peng GL, Tao YL, Wu QN, Zhang Y, He JX (2016). Positive expression of protein chromosome 9 open reading frame 86 (C9orf86) correlated with poor prognosis in non-small cell lung cancer patients. J Thorac Dis.

[79] Chen C, Zhao Z, Tang S, Zhang C (2018). Rab-like protein 1 A is upregulated by cisplatin treatment and partially inhibits chemoresistance by regulating p53 activity. Oncol Lett.

[80] Zhang X, Qi Z, Yin H, Yang G (2019). Interaction between p53 and Ras signaling controls cisplatin resistance via HDAC4- and HIF-1alpha-mediated regulation of apoptosis and autophagy. Theranostics.

[81] Healy FM, Prior IA, MacEwan DJ (2022). The importance of Ras in drug resistance in cancer. Br J Pharmacol.

[82] Zanconato F, Cordenonsi M, Piccolo S (2016). YAP/TAZ at the Roots of Cancer. Cancer Cell.

[83] Yamaguchi H, Taouk GM (2020). A Potential Role of YAP/TAZ in the Interplay Between Metastasis and Metabolic Alterations. Front Oncol.

[84] Bergoug M, Doudeau M, Godin F, Mosrin C, Vallée B, Bénédetti H (2020). Neurofibromin Structure, Functions and Regulation. Cells.

[85] Mo J, Moye SL, McKay RM, Le LQ (2022). Neurofibromin and suppression of tumorigenesis: beyond the GAP. Oncogene.

[86] Tisato V, Voltan R, Gonelli A, Secchiero P, Zauli G (2017). MDM2/X inhibitors under clinical evaluation: perspectives for the management of hematological malignancies and pediatric cancer. J Hematol Oncol.

[87] Kabir MF, Mohd Ali J, Haji Hashim O (2018). Microarray gene expression profiling in colorectal (HCT116) and hepatocellular (HepG2) carcinoma cell lines treated with Melicope ptelefolia leaf extract reveals transcriptome profiles exhibiting anticancer activity. PeerJ.

[88] Kohlmeyer JL, Kaemmer CA, Pulliam C, Maharjan CK, Samayoa AM, Major HJ (2020). RABL6A Is an Essential Driver of MPNSTs that Negatively Regulates the RB1 Pathway and Sensitizes Tumor Cells to CDK4/6 Inhibitors. Clin Cancer Res.

[89] Campbell SL, Philips MR (2021). Post-translational modification of RAS proteins. Curr Opin Struct Biol.

[90] Kano Y, Gebregiworgis T, Marshall CB, Radulovich N, Poon BPK, St-Germain J (2019). Tyrosyl phosphorylation of KRAS stalls GTPase cycle via alteration of switch I and II conformation. Nat Commun.

[91] Bunda S, Heir P, Srikumar T, Cook JD, Burrell K, Kano Y (2014). Src promotes GTPase activity of Ras via tyrosine 32 phosphorylation. P Natl Acad Sci USA.

[92] Jang H, Abraham SJ, Chavan TS, Hitchinson B, Khavrutskii L, Tarasova NI (2015). Mechanisms of membrane binding of small GTPase K-Ras4B farnesylated hypervariable region. J Biol Chem.

[93] Peng Y, Liu J, Inuzuka H, Wei W (2023). Targeted protein posttranslational modifications by chemically induced proximity for cancer therapy. J Biol Chem.

[94] Timofeev V, Samygina V (2023). Protein Crystallography: Achievements and Challenges. Crystals.

[95] Brunger AT, Milburn MV, Tong L, deVos AM, Jancarik J, Yamaizumi Z (1990). Crystal structure of an active form of RAS protein, a complex of a GTP analog and the HRAS p21 catalytic domain. Proc Natl Acad Sci U S A.

[96] Cookis T, Mattos C (2021). Crystal Structure Reveals the Full Ras-Raf Interface and Advances Mechanistic Understanding of Raf Activation. Biomolecules.

[97] Nyiri K, Koppany G, Vertessy BG (2020). Structure-based inhibitor design of mutant RAS proteins-a paradigm shift. Cancer Metast Rev.

[98] Jumper J, Evans R, Pritzel A, Green T, Figurnov M, Ronneberger O (2021). Highly accurate protein structure prediction with AlphaFold. Nature.

[99] Najmabadi H, Hu H, Garshasbi M, Zemojtel T, Abedini SS, Chen W (2011). Deep sequencing reveals 50 novel genes for recessive cognitive disorders. Nature.

[100] Liew J, Amir A, Chen Y, Fong MY, Razali R, Lau YL (2015). Autoantibody profile of patients infected with knowlesi malaria. Clin Chim Acta.

[101] de Goede C, Yue WW, Yan G, Ariyaratnam S, Chandler KE, Downes L (2016). Role of reverse phenotyping in interpretation of next generation sequencing data and a review of INPP5E related disorders. Eur J Paediatr Neurol.

[102] Blondel CJ, Park JS, Hubbard TP, Pacheco AR, Kuehl CJ, Walsh MJ (2016). CRISPR/Cas9 Screens Reveal Requirements for Host Cell Sulfation and Fucosylation in Bacterial Type III Secretion System-Mediated Cytotoxicity. Cell Host Microbe.

[103] Umesalma S, Kaemmer CA, Kohlmeyer JL, Letney B, Schab AM, Reilly JA (2019). RABL6A inhibits tumor-suppressive PP2A/AKT signaling to drive pancreatic neuroendocrine tumor growth. J Clin Invest.

